# Familial Interstitial 6q23.2 Deletion Including Eya4 Associated With Otofaciocervical Syndrome

**DOI:** 10.3389/fgene.2019.00650

**Published:** 2019-07-18

**Authors:** Simone Gana, Angelo Valetto, Benedetta Toschi, Irene Sardelli, Susanna Cappelli, Diego Peroni, Veronica Bertini

**Affiliations:** ^1^Medical Genetics, IRCCS Mondino Foundation, Pavia, Italy; ^2^Section of Cytogenetics, Medicine of Laboratory Department, Santa Chiara University Hospital, Pisa, Italy; ^3^Department of Clinical and Experimental Medicine, Santa Chiara University Hospital, Pisa, Italy; ^4^Section of Pediatric, Department of Clinical and Experimental Medicine, University of Pisa, Pisa, Italy

**Keywords:** EYA4, EYA1, otofaciocervical syndrome, 6q23 deletion, array CGH

## Abstract

We report on a 34-year-old woman and her mother who both have clinical features suggestive for otofaciocervical syndrome (OTFCS), a disorder characterized by a combination of facial dysmorphisms, ear abnormalities with hearing loss, and shoulder girdle anomalies. OTFCS presents overlapping features with branchiootorenal spectrum disorders, including branchiootorenal syndrome and branchiootic syndrome. These disorders have been described as clinically distinct entities, but molecular studies have shown that all the causative genes belong to the Pax-Six-Eya-Dach network (PSEDN). So far, the genetic diagnosis of OTFCS has been performed only in very few cases and involves two genes, EYA1 and PAX1; thus, it is likely that other genes have still to be identified. In the present patient, array CGH analysis showed a 3.7-Mb deletion in 6q23; a smaller 1.9-Mb deletion in the same region was detected in her mother. The minimal overlapping region harbors the *EYA4* gene. The cases here described are interesting, since they all showed the typical clinical features of OTFCS, associated with a deletion in 6q23.2. Even if we cannot exclude the contribution of other genes to the phenotype, EYA4 is a good candidate for OTFCS according to its pattern of expression, its sequence similarity to *EYA1*, and its involvement in PSEDN.

## Introduction

Otofaciocervical syndrome (OTFCS) (MIM#166780, #615560) is a disorder characterized by a unique combination of facial dysmorphisms (long and narrow facies, high arched palate, narrow nose, and narrow mandible), ear abnormalities with hearing loss, and shoulder girdle anomalies (sloping shoulders, low-set clavicles, winged scapulae, and trapezius hypoplasia). Other skeletal anomalies and nasolacrimal duct defects have been frequently reported, whereas cognitive impairment and short stature are seen only in some patients (Fara et al., 1967; Dallapiccola and Mingarelli, 1995; Rickard et al., 2001; Estefania et al., 2006; Mercer et al., 2006).

Even if OTFCS presents overlapping features with branchiootorenal spectrum disorders (BSDs) (Smith, 1993–2018) including branchiootorenal syndrome (BORS, MIM#113650, #610896) and branchiootic syndrome (BOS, MIM#602588, #120502, #608389), these syndromes have been described as clinically distinct entities: phenotypic traits such as facial dysmorphisms and shoulder girdle anomalies were considered specific to OTFCS and were never reported in BORS and BOS, whereas BORS was the only one characterized by functional and structural renal anomalies (Dallapiccola and Mingarelli, 1995).

The more cases reported, the more difficult it is becoming to make a distinction between these syndromes: for example, three cases of OTFCS have been reported to have renal anomalies. Therefore, some authors consider these entities as a unique syndrome with a variable combination of the same clinical signs (Rickard et al., 2001; Estefania et al., 2006; Mercer et al., 2006).

As far as the genetic mechanism is concerned, *EYA1* (Eyes absent, Drosophila, homolog of 1) was the first gene to be identified as causative of OTFCS, BORS, and BOS, highlighting that these syndromes could be due to the same genetic alteration with variable expressivity. Heterozygous pathogenic variants of this gene have been detected in approximately 40% of individuals with a clinical diagnosis of BORS/BOS (Chang et al., 2004; Krug et al., 2011) and in three cases of OTFCS with renal anomalies (Rickard et al., 2001; Estefania et al., 2006; Mercer et al., 2006).

However, other genes, all belonging to the Pax-Six-Eya-Dach network (PSEDN), have been identified; each of them is causative of a more restricted clinical pattern, corresponding to only one of these syndromes (**Table 1**). Heterozygous mutations in *SIX1* (sine oculis homeobox, Drosophila, homolog of 1) and *SIX5* (sine oculis homeobox, Drosophila, homolog of 1) were detected, respectively, in 4% and 5% of individuals with the clinical diagnosis of BORS/BOS (Hoskins et al., 2007; Kochhar et al., 2008), even if the role of *SIX5* in these conditions is still being debated (Krug et al., 2011). More recently, homozygous pathogenic variants in *PAX1* (paired BOX1) have been discovered in three families with OTFCS (Pohl et al., 2013; Paganini et al., 2017; Patil et al., 2018).

**Table 1:** Main clinical features in our patient and in those with mutations in EYA1, PAX1, SIX1, SIX5, and EYA4. Red bars indicate the presence of the clinical signs. NR means “not reported”.

	Cardiac defects	Deafness	Abnormalities of outer ear (including pits/tags)/middle ear/inner ear	Second branchial arch anomalies	Renal anomalies	Lacrimal duct abnormalities	Sloping shoulder	Other cervical/shoulder defects	Long and narrow face	Other facial dysmorphisms
EYA1-related disorders	NR									
PAX1-related disorders	NR				NR		NR	NR	NR	
SIX1-related disorders	NR						NR	NR	NR	NR
SIX5-related disorders	NR					NR	NR	NR	NR	NR
EYA4-related disorders				NR	NR	NR	NR	NR	NR	NR
Our patients	Absent			Absent	Absent	Absent				

We report on a 34-year-old woman and her mother with a clinical phenotype suggestive for OTFCS. Array CGH analysis showed 3.7-Mb deletion in 6q23.1q23.2 in the index case, whereas her mother presented a smaller 1.9-Mb deletion in the same region. Both deletions are flanked by low copy repeats (LCRs). The *EYA4* gene is harbored in the minimal critical deleted region. We hypothesize that its haploinsufficiency may be the pathogenic mechanism underlying OTFCS.

## Case Report

A 34-year-old woman, the only child of a nonconsanguineous couple, was referred to us to define her reproductive risk.

The patient was born *via* a spontaneous, normal pregnancy. No gestational diabetes, uterine malformations, or toxic environment were reported during pregnancy, and delivery was at full term with normal growth parameters. Congenital downward displacement of the shoulders (HP: 0200021) was present at birth; however, no etiological diagnosis was made. The infant’s psychomotor development was normal.

**Figure 1:**
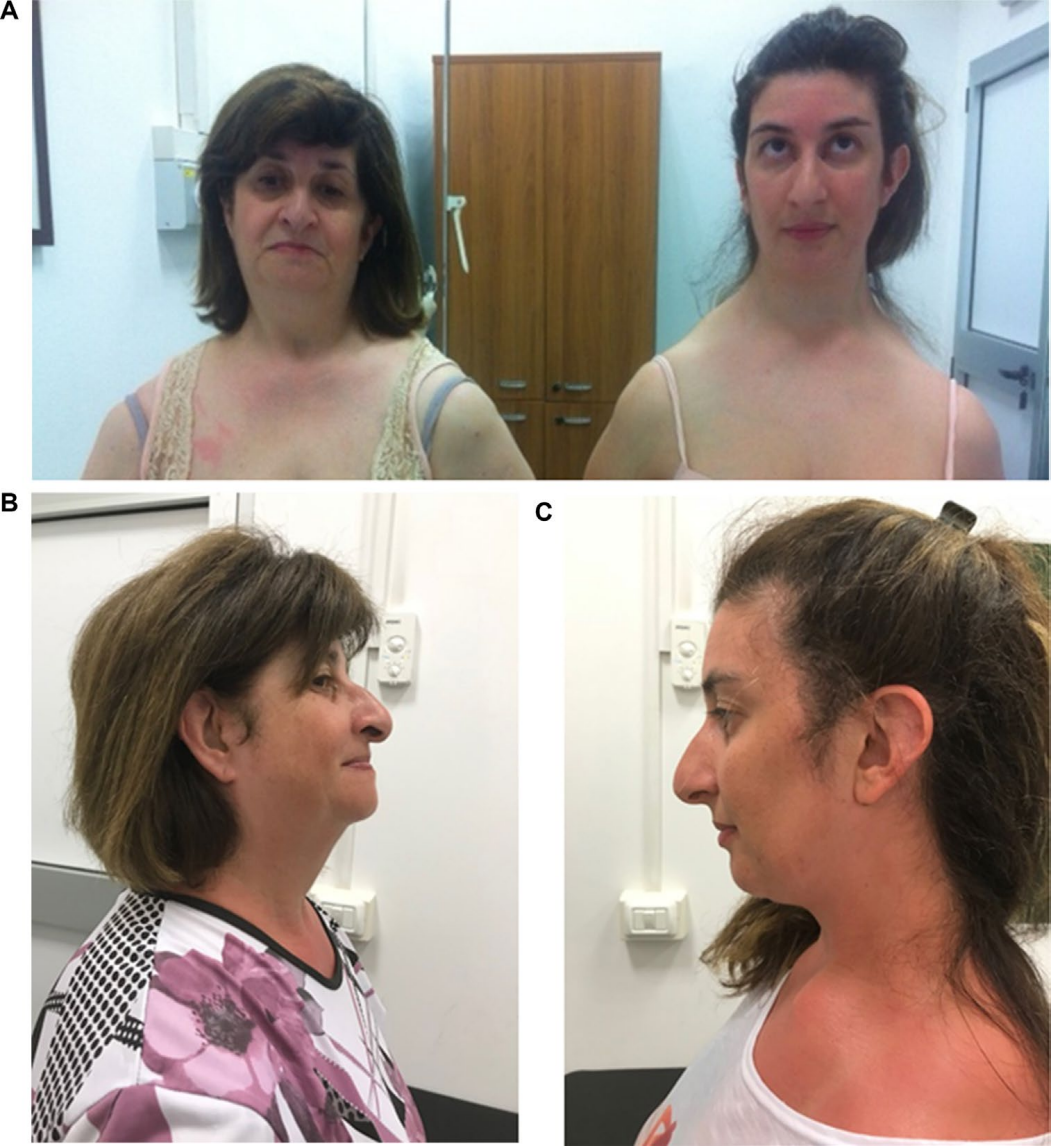
Frontal **(A)** and lateral **(B**, **C)** view of the patient and her mother.

Physical examination at 34 years of age revealed long face (HP: 0000276) with high and narrow nasal bridge (HP: 0000426 and 0000446, respectively), beaked nose (HP: 0000444), high arched palate (HP: 0002705) and narrow mandible (HP: 0012801), prominent ears (HP: 0000411) with large and flat conchae (HP: 0000377), and long neck (HP: 0000472) with asymmetric downward sloped shoulders and clavicles (HP: 0200021 and 0000889, respectively) (**Figure 1**). She was unable to elevate, abduct, and flex her shoulders forward (HP: 0006467). Her stature and cognitive development were normal (the patient was assessed using adaptive behavioral and descriptive clinical criteria).

**Figure 2:**
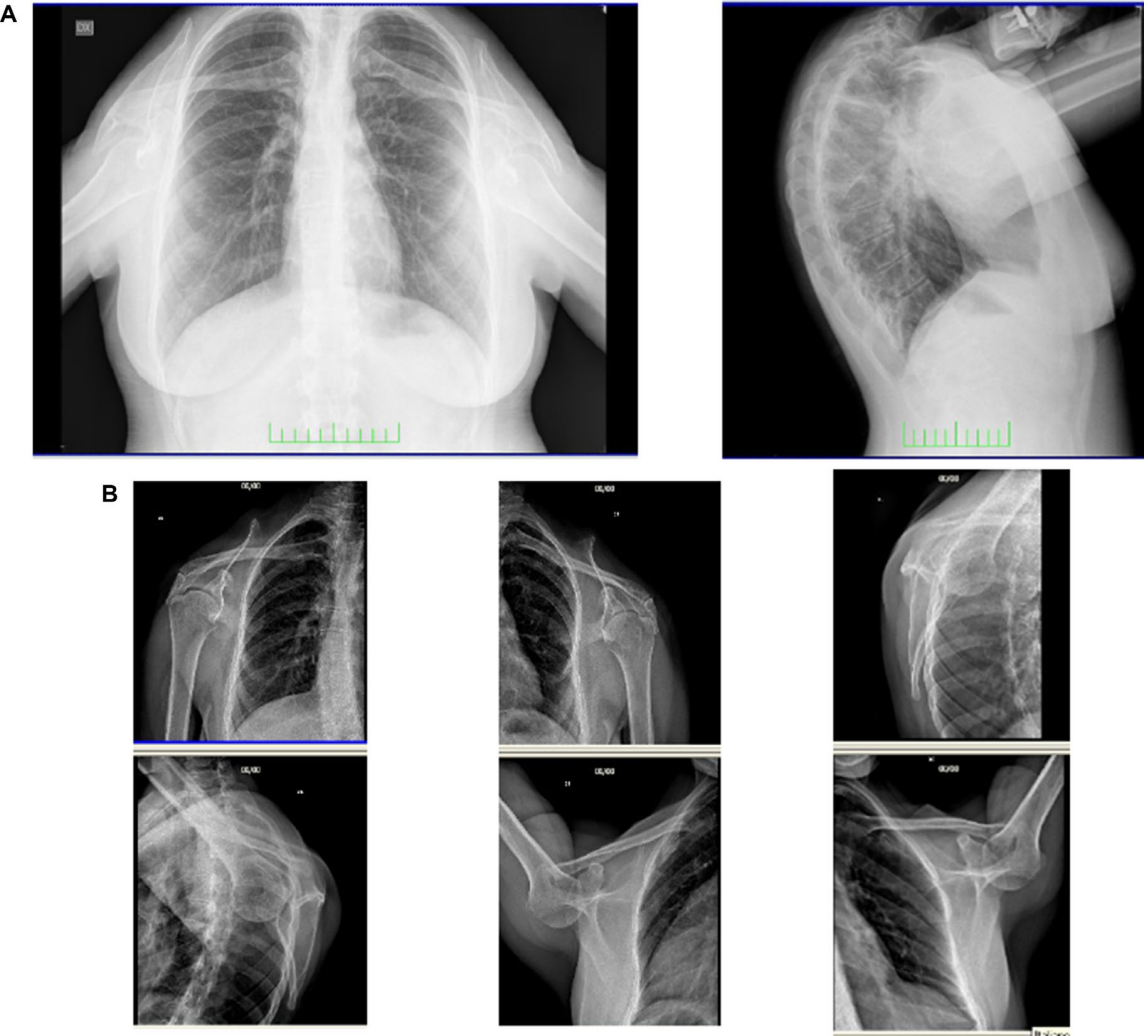
Frontal and lateral radiograph of the patient **(A)**. Small, low, and laterally set scapulae with the glenohumeral joints lower than normal and down-turned clavicles are shown **(B)**.

Chest radiograph demonstrated small, low, and laterally set scapulae (HP: 0000882 and HP: 0200021, respectively), with the glenohumeral joints 10 cm lower than normal (HP: 0200021) and down-turned clavicles (HP: 0000889) (**Figure 2**). Axial and coronal images of shoulder magnetic resonance were normal. Tonal audiometry showed mild bilateral sensorineural hearing loss (HP: 0008619). Ultrasound of the abdomen did not reveal any renal abnormalities. The cardiological evaluation including echocardiogram was also normal.

Her mother had similar phenotypic features: long face (HP: 0000276) with high and narrow nasal bridge (HP: 0000426 and 0000446, respectively), beaked nose (HP: 0000444), and downward sloping shoulders (HP: 0200021) (**Figure 1**). She presented postlingual bilateral, progressive, sensorineural hearing defect (HP: 0008619), which had started at the age of 12. Her cognitive level was normal. She did not present any heart and kidney defects.

Her father had died at the age of 52 due to myocardial infarction (HP: 0001658).

It was not possible to evaluate any other member of the mother’s family, but there was no history of hereditary disorders or congenital malformations.

## Methods and Results

Genomic DNA of the proband and her mother was isolated from peripheral blood by standard methods; DNA from a healthy female subject was used as the control (Agilent Technologies, Santa Clara, California, USA). Two hundred nanograms of genomic DNA from both the patients (test sample) and the control (reference sample) was differentially labeled with Cy5-dCTP or with Cy3-dCTP using random primer labeling according to the manufacturer’s protocol (Agilent). The labeling reactions were applied to the oligo-arrays and incubated for 24 h at 67°C in an oven. The slides were washed and scanned using the Agilent scanner. Identification of individual spots on scanned arrays and quality slide evaluation was performed using the Agilent dedicated software (Feature Extraction, Agilent).

The array CGH was performed on 60K SurePrint G3 Human CGH Microarray (Agilent) that have a 41 kb overall median probe spacing (33 kb in RefSeq genes). The experiments met the “excellent” criteria as determined by the QC report (CytoGenomics software, Agilent). CNVs were identified with CytoGenomics 3.0.6.6. (Agilent), using the ADM-2 (Aberration Detection Method-2) algorithm. The gene content was established by the UCSC Genome Browser (http://genome.ucsc.edu/) (NCBI37/hg19 assembly) and the gene function by RefSeq (https://www.ncbi.nlm.nih.gov/refseq/rsg/).

**Figure 3:**
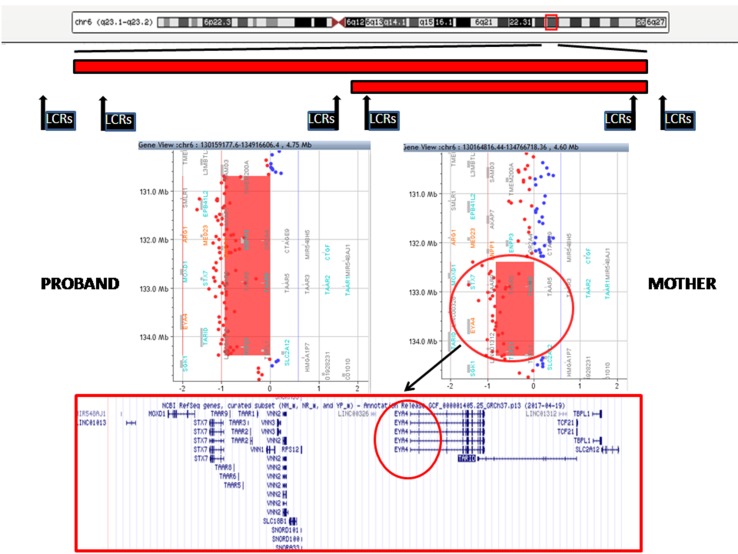
Top: A schematic representation of chromosome 6 with the regions deleted in the proband and her mother (red bars). The position of low copy repeats (LCRs) is indicated by black rectangles. A detail of the array CGH results of the proband (left) and her mother (right) is shown in the middle panels: the genes harbored in the minimal deleted region are reported in the lower panel (*EYA4* is highlighted in black circle).

The proband showed a 3.7-Mb deletion at chromosome 6q23.1q23.2. The proximal breakpoint lay between the oligo in position 130,658,118 (not deleted) and the oligo in position 130,687,781 (deleted); the distal breakpoint was between the oligo in position 134,388,004 (deleted) and that in position 134,464,255 (not deleted). The result was given as arr[GRCh37] 6q23.1q23.2(130687781_134388004)x1. Regarding her mother, she carried a smaller deletion of 1.9 Mb at chromosome 6q23.2: the proximal breakpoint was between the oligo in position 132,279,850 (not deleted) and the oligo in position 132,388,801 (deleted); the distal breakpoint was between the oligo in position 134,310,589 (deleted) and that in position 134,350,097 (not deleted). The final result was given as arr[GRCh37] 6q23.2(132388801_134310589)x1 (**Figure 3**). These deletions are both flanked by low copy repeats (LCRs). Deletion was confirmed by fluorescence *in situ* hybridization (data not shown). The 1.9-Mb minimal overlapping region contains 26 RefSeq genes: 18 coding protein (*MOXD1*, *STX7*, *TAAR1*, *TAAR2*, *TAAR3*, *TAAR5*, *TAAR6*, *TAAR8*, *TAAR9*, *VNN1*, *VNN2*, *VNN3*, *RPS12*, *SLC18B1*, *EYA4*, *TCF21*, *TBPL1*, and *SLC2A1*) and 8 non-coding protein genes (*MIR548AJ1*, *LINC01013*, *SNORD101*, *SNORD100*, *SNORA33*, *LINC00326*, *TARID*, and *LINC01312*). Two of them, *VNN1* (Vanin 1) and *EYA4* (eyes absent, Drosophila, homolog of 4) are present in OMIM (UCSC Database: https://genome-euro.ucsc.edu/).

## Discussion

We report on a 34-year-old female patient with facial dysmorphisms (long face with narrow/beaked nose and narrow mandible), auricular abnormalities, hearing impairment, and sloping shoulders. Her mother presented a very similar facial and shoulder phenotype and exhibited sensorineural hearing loss.

Both these patients show the combination of the specific features (dysmorphic facies, ear abnormalities, hearing loss, and sloping shoulders) originally considered unique and distinctive of OTFCS (Dallapiccola and Mingarelli, 1995); on the other hand, they lack renal defects, reported in BORS, and the second branchial arch anomalies, described in BORS/BOS syndromes (**Table 1**).

So far, the molecular diagnosis of OTFCS has been made only in six patients and involves two genes: *EYA1*, responsible for an autosomal dominant form of OTFCS (OTFCS1, MIM#166780), described in only three cases (Rickard et al., 2001; Estefania et al., 2006; Mercer et al., 2006), and *PAX1*, related to a autosomal recessive form of OTFCS (OTFCS2, MIM#615560), reported in three other families (Pohl et al., 2013; Paganini et al., 2017; Patil et al., 2018).

Array CGH identified two overlapping deletions in 6q23 of 3.7 and 1.9 Mb in the index patient and her mother, respectively (**Figure 3**). Both deletions are flanked by LCRs, which can cause instability in this region by nonallelic homologous recombination (NAHR).

In order to get new insights on the effects of this deletion, we have reviewed the literature data and searched in the public available databases. In DGV (http://dgv.tcag.ca/dgv/app/home), a database collecting CNVs from healthy people, no similar cases were found. We also looked in databases collecting CNVs associated with pathological cases, considering only those deletions overlapping to the minimal deleted region of the present cases, with a single alteration and a phenotype description, we found one case (nssv13638885) in ClinVar database (https://www.ncbi.nlm.nih.gov/clinvar/) and two cases (251447 and 280230) in the Decipher database (https://decipher.sanger.ac.uk/). However, a comparison between these cases and ours is not easy to make, since the extent of the deletion in cases 251447 and nssv13638885 is much bigger than ours (7 and 10.6 Mb, respectively), and the third one (280230) has a very complex and severe phenotype, including global developmental delay together with multiorgan problems, which could be the results of other cryptic alterations.

The 1.9-Mb region encompasses 26 RefSeq genes (**Figure 3**), and two of them, *VNN1* and *EYA*, are referenced in the OMIM database. All genes reported only on RefSeq genes do not seem to be relevant candidates; a contribution of *TCF21* to sensorineural hearing loss was assumed, but it has not been confirmed (Arbustini Eloisa et al., 2005; Vona et al., 2014). A role of *VNN1* in the patient’s phenotype could be ruled out, since VNN1 is implicated in plasma concentrations of high-density lipoprotein cholesterol (Goring et al., 2007). By contrast, *EYA4* appears to be a good candidate for OTFCS.

*EYA4* belongs to the eyes absent gene family that has a high degree of conservation through animal evolution, ranging from insects to humans (Ikeda et al., 2002; Kozmik et al., 2007).

Vertebrates encode four EYA proteins (EYA1–4) that are characterized by a conserved C-terminal 271-amino-acid domain (eya-HR) and a poorly conserved N-terminal domain (eya-VR NTD), which ranges in size between 266 and 320 amino acids. The HR is involved in protein interaction and has a tyrosine phosphatase domain; the VR presents a transcriptional activation and threonine phosphatase domains. Thus, Eya proteins are not purely transcriptional activators, but they combine several biochemical activities in a single polypeptide (Tadjuidje and Hegde, 2013). EYAs have been associated with a wide range of biological processes, including cell proliferation, migration, angiogenesis, DNA damage repair, innate immunity, and photoperiodism (Tadjuidje and Hegde, 2013).

A survey of the expression profile of the human transcripts using the UniGene database reveals that EYAs are present from the embryoid body stage through adulthood. Although each gene has a unique expression pattern, there is wide overlap (Miller et al., 1997; Depreux et al., 2008; Tadjuidje and Hegde, 2013).

All the four EYA proteins are components of a conserved regulatory network that is often referred to as PSEDN to better reflect the genes/proteins involved. This network plays a key regulatory role in the early development of multiple organs (Ikeda et al., 2002; Kozmik et al., 2007).

So far, all disease-genes identified as causative of OTFCS and BORS/BOS belong to this molecular network (Rickard et al., 2001; Chang et al., 2004; Estefania et al., 2006; Mercer et al., 2006; Hoskins et al., 2007; Kochhar et al., 2008; Krug et al., 2011; Pohl et al., 2013; Paganini et al., 2017; Patil et al., 2018). Therefore, these conditions could be subsumed under the umbrella term of PSEDN-related disorders.

Taking into account its pattern of expression, its sequence similarity to *EYA1*, and its involvement in PSEDN, *EYA4* could be an excellent candidate for the clinical features of OTFCS present in our patients.

To date, most patients harboring *EYA4* mutations present with autosomal dominant progressive sensorineural hearing loss; dilated cardiomyopathy has also been reported (Wayne et al., 2001; Schonberger et al., 2005; Abe et al., 2018). However, only a limited number of papers regarding mutations in this gene have been published, and the clinical effects are still to be fully elucidated. Our patients do not have cardiac problems, and they present many other clinical features not reported before in *EYA4* mutation cases. It is worth noting that the patients so far described have point mutations in the coding sequence, whereas this large deletion could also harbor also regulatory elements that could have additional effects on the phenotype.

According to molecular studies, OTFCS and BORS/BOS can be considered as a unique syndrome, where the different clinical signs may be due to a variable expressivity of EYA1. On the other hand, *PAX1*, *SIX1*, and *SIX5* mutations do not seem to have such a variable phenotypic expression, but each of them is responsible for only one of these syndromes. Therefore, the clinical differences between PSEDN-related disorders may depend on the gene involved.

In view of this, the cases here presented are interesting, since they showed all the typical clinical features of OTFCS associated with a deletion in 6q23.2, which harbors a gene belonging to PSEDN.

## Concluding Remarks

In summary, we have presented a family with autosomal dominantly inherited OTFCS and a 6q23.2 deletion segregating with the phenotype and harboring *EYA4*. This gene could be an excellent candidate for OTFCS according to its pattern of expression, its sequence similarity to *EYA1*, and its involvement in PSEDN, even if further cases are needed to completely elucidate this link. Identification of new genes causative of PSEDN-related disorders will be useful for a more accurate nosology of this group of disorders: overlapping clinical features can be split or lumped together according to the causative genes belonging to the same molecular pathway.

## Ethics Statement

This study was carried out after written informed consent of all the subjects was received. The analyses performed did not require the approval of our hospital’s ethical committee.

## Author Contributions

SG, AV, and VB participated in the design of this study.

VB, IS, and AV performed the array CGH and the CNV gene content analyses.

SG, VB, and AV contributed to writing the manuscript.

SG, BT, DP, and SC performed the clinical investigations and critically revised it.

All authors have read and approved the final version of this manuscript.

## Funding

The authors have no financial disclosures to make.

## Conflict of Interest Statement

The authors declare that the research was conducted in the absence of any commercial or financial relationships that could be construed as a potential conflict of interest.
